# Suggestions for establishing a sustainable risk communication platform for carcinogenic factors

**DOI:** 10.4178/epih/e2014034

**Published:** 2014-12-09

**Authors:** Keeho Park, Yong-Chan Kim, Youngho Kim, Meeyoung Cha, Woon Heui Han, Dae-Kyu Oh

**Affiliations:** 1 Cancer Policy Branch, National Cancer Center, Goyang, Korea; 2 College of Communication, Yonsei University, Seoul, Korea; 3 College of Education, Korea University, Seoul, Korea; 4 Graduate School of Culture Technology, Korea Advanced Institute of Science and Technology, Daejeon, Korea; 5 Media Laboratories, Yonhap News Agency, Seoul, Korea; 6 Department of Preventive Medicine, Gachon University School of Medicine, Incheon, Korea

## INTRODUCTION

Given the steady increase in the number of cancer patients and media reports on controversial carcinogens related to occupation and lifestyle, people’s interest in cancer is growing. Since the Korean government pledged to overcome cancer, establishing its “Conquering Cancer 10-Year Plan” in 1966, the “Conquering Cancer” policy has achieved an increased cancer survival rate; however, the number of cancer patients is constantly increasing, due to the factors of aging, lifestyle, and environment. Of those people who achieve the average life expectancy (77 years for men and 84 years for women), it is estimated that two out of five men (38.1%) and one out of three women (33.8%) will suffer from cancer [1]. Under these circumstances, recent media reports on the carcinogens found in everyday products such as drinks, bags, and sportswear—as well as an announcement by the International Agency for Research on Cancer (IARC) about the carcinogenicity of diesel combustion—have caused national repercussions and a debate about cancer risks. Individuals’ ability to communicate about various health risks through a variety of media, digital devices, and widespread social network services (SNS) has enabled social discourse and arguments to develop at a much faster rate than would have been the case in the age of traditional media.

Topics addressed during various debates about carcinogens have ranged from the impact of short-term and local factors to the damage caused by longer-term and global factors. It is common for many risks faced in modern society (the “risk society”) to develop uncontrollably, even if they start as short-term or disconnected risks. Risk communications in modern society can be described as a long-term, complicated form of communication, in which various agents are intricately connected with one another—not the simple, one-way communication style of a single agent (for example, the government) communicating with the general public. This trend is increasingly reinforced through the development of new media such as the Internet, mobile devices, and SNS. For this reason, communications related to carcinogenic risk factors may evolve to an uncontrollable level because of one-sided media coverage and people’s excessive production of SNS messages. The most significant factors include the following: the gap between the risk perceptions of experts, government, and the general public; the general public’s lack of professional knowledge; the understanding imbalance between the expert group and the general public. All of these factors increase people’s distrust of information delivery and amplify their anxiety about risk factors. A process of agreement through mutual communication is therefore needed, essentially because social discourse about risk issues (being closely connected to socially formed value issues) cannot be created solely on the basis of objective, expert technical judgments.

To establish national strategies for communicating carcinogenic risk factors to the public, while building a virtuous system of communication agents who can speak on behalf of the public, industry, media, and governments (including public institutions), researchers need to explore the following considerations: first, the ways in which information related to carcinogenic factors will flow; second, when and how people’s awareness, knowledge and attitudes toward carcinogenic factors are formed, together with how they change and respond; and third, when and how agents of communication at the national level should respond.

The US has established an extensive survey data network called the Health Information National Trends Survey (HINTS), which provides data on how people use health facts, as well as information on various cancers, health communications, health service and Internet use, social networking, smoking, doctor-patient communications, sports, and nutrition. The HINTS was established by the Health Communication and Informatics Research Branch of Cancer Control and Population Sciences, an organization affiliated with the National Cancer Institute (NCI). NCI established HINTS because the rapidly changing environment for health communications led to an awareness of the monitoring problem. There was also a consensus on the need to examine people’s awareness of, attitude toward, and knowledge about various types of cancer, as well as their understanding of the environments available to effectively manage cancer. If surveys are conducted and analyzed continuously, it will become possible to systemically analyze people’s information data usage patterns and problems, as well as health communication differences in relation to types of cancer and the impact of the media on people.

Developed countries actively conduct studies using big data to analyze and predict social issues in various ways. It is acknowledged that big data analysis can help detect developing risk issues, negative public opinions, and controversies, reducing the social costs of responding and acting to improve people’s quality of life and solve worldwide problems. One typical example involves Google, the global search engine, which, in 2009, calculated the frequency of search queries related to the flu, predicted flu activities in many countries around the world, and made its service available to users. In addition to these structured analyses, unstructured SNS-driven big data analysis has emerged as a central issue that currently influences various aspects of the public and private sectors. A study by Sadilek et al. [2] on big data processing showed how Twitter was used to identify disease factors, track the path of infection between individuals, and predict the spread of infectious diseases. Global positioning system (GPS)-tagged tweets (approximately 4.5 million), a subset of the 16 million tweets collected, were analyzed to extract those containing disease-related terms. From these, it was possible to derive a correlation between places where people with a disease factor were co-located, the size of their social networks, and their likelihood of contracting a disease.

In the meantime, a study by Christakis & Fowler [3] that had been collecting data for 32 years, persuasively argued that the prevalence of obesity might partly be influenced by one’s social network. Between 1971 and 2003, the body mass index of 12,067 people in a specific area were collected and analyzed to assess whether their weight changes were correlated with the weight changes of friends, family members, neighbors, and other people around them. These studies indicate the need to consider SNS data in assessing potential health risks in the society.

## BODY

### Distribution of health information through the press and media; changes in the distribution environment

In South Korea, the majority of people obtain health-related information through the mass media, while most young people obtain health-related information via the Internet. Now that SNS have overcome the traditional media, which previously dominated agenda setting, anyone can own a medium and express one’s opinion. As communication channels have become more diverse, people freely share information on diseases and treatments, even in the healthcare environment. This phenomenon is called the *Medicine 2.0*; it has attracted much attention in foreign countries and been used to develop practical services such as Ask a patient (http://www.askapatient.com/) and Google Flu Trends (http://www.google.org/flutrends/). At the same time, information distribution through SNS has caused many problems, including online information credibility issues, discrepancy between press reports and SNS information on particular topics, and the debate of the pros and cons, inaccurate information, and myths that can mislead people searching for health-related information. For this reason, a close monitoring of health-related information sharing and discourse is urgently needed. For SNS platforms that share information through Application Programming Interfaces, open media monitoring is relatively easy, although closed media data is restricted. For national issues or discourses, it is possible to exploit the fact that topics discussed in open and closed media are often correlated.

### Research on disease and the impact of health-related human environments (space analysis)

Space analysis analyzes an individual’s health-related indicators, behavioral data, and various environmental factors spatially, evaluating their relevance. Representative research topics include the impact of environment on diseases, regional health variations, and the spatial mismatching of health services and access to health services. Because the socio-demographic characteristics of a city have been differentiated according to space, communication and media are presented differently in different regions [4]. A disease does not simply reflect the state of one individual’s health, but also the interaction of various socio-demographic variables, including personal characteristics, the impact of society and the natural environment, and social relationships and institutional arrangements; disease can be explained as a spatial phenomenon that manifests as a combination of these factors [5]. Modern communication media possess a spatial attribute [6]. As geographic information via online mapping is incorporated into media, and location information is added to SNS, network services and other media include spatial information features. The combination of media and spatial information is gradually developing, while interactions between space and communication are expanding. For this reason, social communications about carcinogenic risk factors cannot explained by one or two methodologies and theories, but require the analysis of many other factors, including an individual’s socio-demographic variables and the spatial environment. Determining the impact of various spaces and the surrounding environment requires an analysis of an individual’s movement process and path, to determine how these affect risk communication.

### Establishing and managing systems of carcinogen-related big data

As existing carcinogen-related data are scattered across a variety of different institutions, it is difficult to identify correlations between the data. The carcinogen-related data in various institutions should be collated and analyzed so that useful results can be derived. In other words, statistical data related to carcinogenic factors that are held in medical and health-related government agencies and private medical institutions should be aggregated. In addition, for effective communication management, the comprehensive data relating to media (which produces and distributes messages) and individuals (who produce and consume messages) should be amalgamated. During this process, the structured data possessed by medical institutions and government agencies and the unstructured data disseminated by the Internet, SNS, newspapers, and radio and television broadcasts, should be treated as legitimate subjects for media analysis. Communications related to carcinogenic factors should be analyzed visually and spatially, so that their diffusion and spread can be related to the communicators’ social hierarchy and age, with the aim of supporting smooth, effective communication.

### Integrated and continuous panel research

Identifying precisely how the public understands cancer-related information and investigating promptly how people perceive and cope with problems will require the establishment of a public awareness panel research system focusing on carcinogenic causes, cancer prevention, and people’s behavior. Such a system could provide follow-up after changes in public awareness, knowledge and behavior, implementing immediate corrective actions whenever misconceptions arose or inadequate information was distributed.

**Figure 1.**
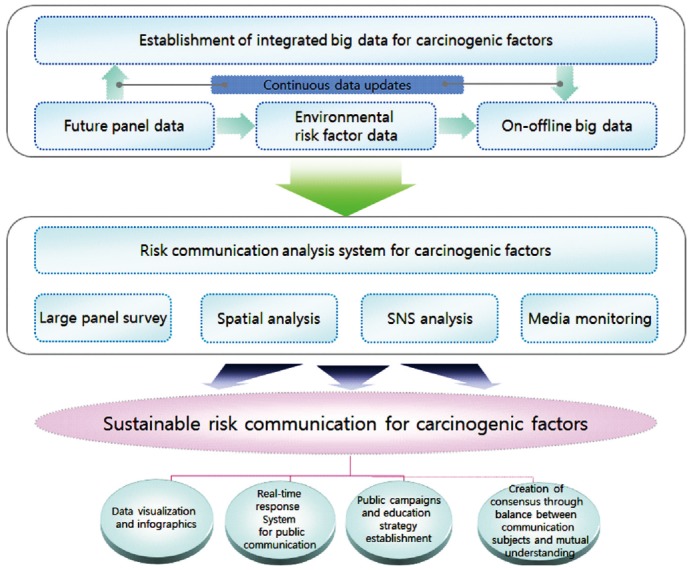
Sustainable risk communication platform for carcinogen risk factors. SNS, social network services.

A sustainable risk communication platform that enables a serial process of data collection, storage, management, analysis, result production, and information delivery to the public should be established (Figure 1) to meet existing needs and integrate big data that relates to carcinogenic factors by applying the latest interdisciplinary methodologies. Establishing this integrated platform and managing it as a unified system through a government-designated institution should contribute to designing timely policy communication measures that deal with the context and health concerns of carcinogen risk factors, establishing a response strategy that meets situational needs, supporting itemized and optimized decision making.

The tasks required to establish a sustainable risk communication platform for carcinogenic factors are presented as below.

#### Establishing and managing integrated big data

##### National Cancer Registry data

The National Cancer Registry data will support the systemic management of cancer and cancer research. As the geographical distribution of cancer patients is one of the most important elements in an epidemiological analysis of cancer, a geographical analysis of cancer patients using the National Cancer Registry data will provide important data on carcinogenic factors.

##### Demographic data

Demographic analysis is based on humanistic-social phenomena and takes into account population, population density, population movements, gender ratio, and age. Established demographic data can be applied in various fields and used to identify the attributes of space. The socio-economic characteristics of a particular area (demographic characteristics expressed in space) should be examined along with demographic data. In addition, spatial analysis should also make use of socio-economic information such as occupation, income, education, and taxes. The Ministry of Transportation provides various land- and transportation-related statistical data, including a spatial information service, national spatial integration, an electronic land service, and a national geographic information distribution service and network that provides maps via the geographic information system (GIS) service. In particular, information about apartments, standard official land prices, and real estate data can be used to estimate the regional income quintile. It will also be necessary to collect data and statistical screening information from the National Statistics Office, the Ministry of Transportation, and the Ministry of Education, as well as from reports issued by population analysis institutions.

##### Natural environment data

Natural environment data comprises all physical factors affecting human life, including behavior and lifestyle. The terrain, with its altitude, slope, undulation, soil and land cover conditions, and climatic factors (average annual temperature, temperature ranges, precipitation, wind speed, and humidity) are categorized as environment data. As individual elements of the natural environment act in combination, a comprehensive understanding is more important than a detailed analysis of individual phenomena. The National Geographic Information Institute manages and sells digital maps detailing land characteristics and the status of land use, as well as aerial photographs and videos. The Ministry of the Environment provides data on land cover conditions, surface water quality, air pollution, and waste generation through its affiliated institution, the Environment and Geographic Information Service. In particular, users of public institutions have access to a spatial information source data download service that collects natural environment data from the Ministry of the Environment. The National Weather Service offers an analysis of monthly climate statistics, including average annual temperature, the annual temperature range, precipitation, wind speed and direction, humidity, evaporation, frost days, and foggy days, along with maps detailing weather and climate. Statistical data on climate, weather factors, and meteorological location points are provided by the National Weather Service, making it possible to conduct secondary processing using collected data. The conversion of these natural environmental data to a format that allows for comprehensive analysis makes it possible to conduct spatial analysis research on cancer, using demographic and other spatial elements.

##### Data on environmental risk factors related to cancer

If environmental carcinogens are naturally present or if there is a facility where carcinogens are thought to have been intentionally released, researchers must build location point data using geographical information, such as addresses. In South Korea, there are no standard definitions or lists of environmental carcinogens. It is possible to extract some mismatched information about carcinogens and environmental carcinogens under the Occupational Safety and Health Act of the Ministry of Labor and the Hazardous Chemicals Management Act of the Ministry of the Environment. If it is deemed necessary, it is possible to use lists of carcinogens developed by foreign institutions such as the WHO IARC. Because the motivation for recording location information on environmental carcinogens as point data and establishing a database generally reflects suspicions about carcinogen-related facilities, follow-up is required to improve the reliability of large data.

##### Information on types of industrial facilities, carcinogenic emissions, and impacts should be entered in the built point data, which can be used in carcinogen-related analyses.

Because carcinogenic environmental risk factors change over time, continuous updating is necessary, through the use of designated data collection periods. After data on environmental carcinogenic risk factors have been collected, the impact of the related environment or facility on the surrounding population can be evaluated through an analysis of the diffusion rate, influence, and location point where carcinogen has been discharged.

##### News monitoring of carcinogenic factors

It is very important to keep track of the way in which media reports about carcinogenic factors are made. This is because the public’s awareness of carcinogenic factors is influenced by media reports to a significant extent. The results of news monitoring are integrated into other data (for example, SNS social discourse data), and used to trace and analyze the path and spread of social discourse on carcinogenic factors. Monitoring the news may also provide important data for building an integrated risk management system in which the media itself is important subject of study.

The media outlets analyzed are mainly typical news media (newspapers and TV and radio stations) in Seoul and other large cities, the top ten major daily newspapers, and prescreened and selected broadcasters in Seoul and other cities (in addition to KBS, MBC, and SBS). Data collection is carried out using the Korea Press Foundation’s media publicity database; data screening involves an exhaustive search for articles that include the keywords “carcinogen,” carcinogen report,” or “carcinogen information.” Key items for analysis include media coverage volumes, reporting frames, reporting attitudes, and the orientation and quality of reporting.

##### Analysis of discourses on social network services relating to carcinogenic factors

To collect and organize a large volume of data (various social discourses) related to carcinogenic factors and users on SNS, it is necessary to determine the methods, time period, and scope for optimal social network data mining. After inputting the mass of collected SNS data into the database for fast processing and analyzing, words related to carcinogenic factors are selected and revised appropriately. Evidence-based carcinogenic factors and related SNS search terms are also additionally selected. Only carcinogen-related terms and user information are extracted from the SNS data, and characteristics the users’ social networks are analyzed using a variety of quantitative analysis methods. Comparisons of quantitative indicators such as density, centrality, clustering coefficients, and structural holes in the social network can reveal changes in user references to carcinogenic factors and changes in the way that social networks of users spread false information about carcinogenic factors, depending on the characteristics of each factor. We also explore how the social network-related quantitative indicators of users who mention cancer on SNS change depending on the type of cancer. After completing the quantitative analysis, the emotional quality of carcinogenic-related social discourse on SNS is analyzed using the natural language analysis method. From this research, the following qualitative research topics may be extracted:

- How do people emotionally react to news or information about various carcinogens and carcinogenic factors?
- How will these feelings change if the information turns out to be inaccurate—or is verified through scientific experiment?
- When new information on a carcinogenic food product is presented, how quickly will people’s feelings change into a particular emotional state?
- Classical methods (reading, understanding, and analyzing SNS contents directly) can complement computer-aided natural language analysis in overcoming accuracy problems. In particular, a variety of qualitative manual analysis can be performed at low cost using a service called Mechanical Turk of Amazon, which has been actively used in many qualitative studies in recent years, in parallel to manual analysis. In South Korea, there is a similar small-scale, survey-based service; wiki-based topic analysis is also well suited to South Korea, given its high Internet penetration and participation rates.

##### Big data-based spatial analysis utilizing geographic information system

Spatial data on people’s social environments take account of land use, traffic routes, subways, buses, parks, hospitals, and educational institutions. Natural environmental data include streams, mountains, and the green belt. Basic statistical information covers administrative districts, businesses, employees, demographic characteristics, transfer information, and local taxes. When necessary, a survey of a particular region is conducted. A survey area and target group that reflects the gender and age balance of the local population are assigned, and a survey application is installed in responders’ smart phones. After the survey, the correlation between regional differences and the survey results, as well as spatial distribution and the socio-demographic environment are analyzed. The smart phone application identifies the spatio-temporal patterns of movement of survey respondents, transmitting their location information through smart phones to a server at predetermined time intervals, thus enabling the server to record the location of individuals over time. Classified by gender, age, and, occupation, a user’s activity area (defined by hours of work, leisure time, and household residence time) is assigned to a cluster, so that the contents of risk-related communications can be compared. The individual’s exposure to environmental factors can be analyzed by studying his or her moving path. Such analyses, using GPS/GIS technology, enable a precise evaluation of environmental, human, and social factors.

##### Panel survey on public awareness of carcinogens

It will be important to establish a public awareness panel survey system to correctly assess the public’s understanding of cancer-related information and explore how people perceive risk and cope when a problem arises. Such a system would reveal changes in public awareness, knowledge, and behavior related to carcinogenic factors over time, making it possible to take immediate corrective actions to counteract misconceptions and the distribution of misleading information.

The survey will evaluate the public’s understanding of carcinogenic factors, and assess attitudes and beliefs, motivations for seeking information, behavior patterns, information acquisition sources, and information sharing patterns. It will reveal the following information: the degree of public knowledge of carcinogenic factors; which personal, social networking, and regional factors make a difference in the awareness of carcinogens; who are most susceptible to information about carcinogenic factors, how quickly rumors spread; the relationship between understanding carcinogenic factors and taking action to prevent cancer; and the relationship between misperceiving carcinogenic factors and taking action to prevent cancer. This will become an important resource for those seeking to protect vulnerable people from false information and rumors related to carcinogenic factors.

##### Monitoring and conducting in-depth interviews with participants in social network services discourses related to carcinogenic factors

We monitor and conduct in-depth interviews with users who participate in online SNS discourses related to carcinogenic factors and carcinogens. Once user IDs have been obtained through SNS data collection, the SNS usage and responses to other user issues are examined over a period of time, and compared to previous carcinogen-related reactions. In addition, people who have posted or spread incorrect information about carcinogenic factors and carcinogens are monitored, and their general SNS usage patterns observed and compared. After analyzing issues from various angles (by groups and time periods) we interview a sample group of users before finally interpreting the results. This provides data that can’t be found through SNS content analysis alone, such as the offline sources used for information, users’ motivation for spreading incorrect information, and the medical expertise of users who participate in discourses and information spreading.

##### Data visualization and infographic implementation for effective risk communication

Recently, the value of the infographic has been gradually spreading through the media, business and public institutions (government, organizations, and schools). Public institutions are upgrading their public services and turning press releases into a form of infographic as part of information delivery. Data visualization, as a method of presenting big data analysis results, is essential; visualization and infographic implementation can be a persuasive tool for effectively communicating with the public about socially controversial risk factors and issues.

## CONCLUSION

A sustainable risk communication platform for carcinogenic factors may help prepare short-term and long-term response measures to deal with the flow of public opinion on carcinogenic issues. In the short-term, it may be used to establish a public relations system for communicating with the general public when a particular issue related to carcinogenic factors occurs. In the long-term, it will accumulate comprehensive data for cancer-related infodemiology [7] in South Korea. In addition, this platform is expected to contribute toward establishing a technical infrastructure that can overcome methodological limitations among the disciplines through interdisciplinary fusion research.
