# Supplementary material for: Suggestions for establishing a sustainable risk communication platform for carcinogenic factors
Source: Epidemiol Health. 2014 Dec 9;36:e2014034. doi: 10.4178/epih/e2014034 (PMC4282309; doi:10.4178/epih/e2014034)
Supplement: Supplementary file 1 [file epih-36-e2014034-supplemetaldata.pdf]

제목

발암성 위험 요인 관련 지속 가능한 리스크 커뮤니케이션 플랫폼 구축을 위한 제언

서론

최근 암환자가 꾸준히 증가하고 직업, 생활관련 발암물질 논란에 대한 미디어 보도가 증가함에 따라 암에 대한 사람들의 관심 또한 증가하고 있다. 1996년, 정부가 암 정복을 선언하고 ‘암 정복 10개년 계획’을 수립한 이후, ‘암 정복’ 정책을 통해 암에 대한 생존율을 높이는 성과를 달성하였으나, 고령화, 생활습관, 환경 등의 영향으로 암환자는 지속적으로 증가하고 있으며, 평균수명(남 77세, 여 84세)까지 생존할 경우, 남자는 5명중 2명(38.1%), 여자는 3명중 1명(33.8%)이 암에 걸릴 것으로 추산되고 있다 [1]. 이러한 가운데 최근 음료수, 가방, 운동복 등 생활용품에서 발견되는 발암물질에 대한 언론 보도 및 디젤 연소물의 발암성에 대한 국제암연구소의 발표 등은 암 위험에 대한 다양한 국민적 반향을 일으키기에 충분했다. 이러한 다양한 건강 위험에 대한 사람들간의 소통은 다양한 매체를 통해 이루어지게 되는바, 각종 디지털 기기와 소셜네트워크서비스(Social Network Service, SNS)의 확산으로 인하여 과거의 전통적 미디어 시대에 비해 훨씬 빠른 속도로 다양한 사회적 담론 및 갈등을 형성할 수 있게 되었다.

이러한 리스크 커뮤니케이션의 대상은 단기적이고 국지적인 요인에 의해 발생하는 것에서부터 중장기적이고 전지구적인 요인에 의해 발생하는 것까지 다양하다. 하지만 ‘위험사회’의 특징을 갖고 있는 현대사회에서 우리가 겪는 리스크들 중 많은 것은 처음엔 단기적이고 단편적으로 시작했다 하더라도 시간의 흐름에 따라 통제 불가능할 정도의 규모로 커지는 경우가 비일비재하다. 그래서 현대 사회의 리스크 커뮤니케이션은 하나의 주체(예를 들어 정부)가 대중을 상대로 하는 단순하고 일방적 방식의 커뮤니케이션이라기 보다는 다양한 커뮤니케이션 주체들이 서로 유기적으로 연결된 상태에서 진행되는 장기적이고 복잡한 형태의 커뮤니케이션이라고 할 수 있다. 이러한 경향은 인터넷, 모바일, SNS 등 뉴미디어의 발전으로 더욱 강화되고 있다. 예를 들어, 발암성 위험 요인 관련 리스크 커뮤니

케이션의 경우 미디어의 일방적인 보도, SNS 등을 통한 사람들의 과도한 메시지 생산으로 인하여 통제 불가능한 수준에 이르기도 한다. 이러한 문제의 가장 큰 원인은 전문가, 정부 그리고 일반대중의 위험 인식 차이에서 비롯되는데, 대중의 전문적인 식견 부족, 전문가 집단과 대중 간 이해의 불균형으로 인해 정보전달에 대한 불신을 야기하며, 국민들 사이에서 위험요인에 대한 불안감을 증폭시키게 된다. 그러므로, 위험 이슈에 대한 사회적 담론은 전문가의 기술적이고, 객관적인 판단에 부합하게만 형성될 수 없고, 사회적으로 형성된 가치문제와 밀접하게 연결되어 있기 때문에 상호 커뮤니케이션을 통해 서로 간 합의의 과정이 필수적으로 요구된다고 볼 수 있다.

따라서, 발암성 위험 요인과 관련한 국가적 리스크 커뮤니케이션 전략을 세우고 대중, 산업, 미디어, 정부(관련 공공기관 포함)로 나뉘는 발암성 위험 요인 관련 커뮤니케이션 주체들의 선순환적 커뮤니케이션 체계를 구축하기 위해서는 첫째, 발암성 위험 요인과 관련한 정보가 어떠한 방식으로 흘러가게 되는지, 둘째, 발암성 위험 요인과 관련한 사람들의 인식이나 지식, 태도가 언제, 어떻게 형성되어 어떠한 변화 과정을 거쳐 어떻게 반응하는지, 셋째, 국가 차원에서의 커뮤니케이션 주체가 언제, 어떻게, 어떠한 방식으로 대응해야 하는가에 대한 연구가 필요하다고 하겠다.

미국의 경우, Health Information National Trends Survey(이하 HINTS)라는 광범위한 서베이 데이터망을 구축하여 국민들이 어떠한 방식으로 건강정보를 이용하는가부터 다양한 종류의 암 관련 지식, 헬스 커뮤니케이션, 헬스 서비스 및 인터넷 사용, 사회 네트워크, 흡연 여부, 의사-환자 커뮤니케이션, 운동과 영양 등에 대한 정보를 제공하고 있다. HINTS는 미국 국립암연구소(National Cancer Institute. 이하 NCI)의 산하조직인 Cancer Control and Population Sciences (DCCPS)의 Health Communication and Informatics Research Branch (HCIRB)에 의해서 만들어졌다. NCI가 HINTS를 구축하게 된 것은 헬스 커뮤니케이션의 환경이 빠르게 변화함에 따라 이에 대한 지속적인 모니터링이 필요하다는 문제의식이 있었으며, 암을 효과적으로 관리하기 위해서는 다양한 종류의 암에 대한 사람들의 인식, 태도, 지식과 더불어 현재 사람들의 건강상태, 그들을 둘러싼 다양한 환경에 대한 파악이 필요하다는 것에 공감하였기 때문이었다. HINTS와 같은 대규모 서베이를 통해 커

뮤니케이션의 수용자에 대한 광범위한 분석이 지속적으로 이루어진다면, 사람들의 건강정보 이용패턴과 문제점 뿐만 아니라 암의 종류에 따른 헬스 커뮤니케이션의 차이와 사람들에게 미치는 미디어의 영향력에 대한 체계적인 분석이 가능하게 된다.

선진국에서는 빅데이터를 이용해 사회적 이슈에 대해 다양하게 분석하고 예측하는 연구를 활발하게 진행하고 있다. 이는, 빅데이터 이슈 분석을 통해 위험 사안을 비롯한 다양한 찬반 논쟁, 부정적 여론 등의 징후를 사전에 감지하고, 대응하기 위한 사회적 비용을 절감할 수 있으며, 나아가서는 삶의 질 향상과 인류적 문제해결 수단으로 활용할 수 있다는 인식이 자리잡고 있기 때문이다. 이와 관련한 보건 이슈의 대표적 사례로, 세계적인 웹 검색 사이트인 구글에서 2009년 독감과 관련된 검색어의 빈도를 계산하여 전 세계 여러 국가, 지역에서 독감 유행 수준을 예측하고, 독감확산 현황을 알아볼 수 있도록 서비스하고 있다. 이러한 정형화된 빅데이터 분석과 더불어 비정형화된 SNS상의 빅데이터 분석도 중심이슈로 떠오르면서 현재 공공 및 민간 부문의 다양한 영역에서 영향력을 발휘하고 있다. 건강 관련 트위터의 빅데이터 처리로 분석을 시도한 Sadilek 등의 연구 [2]는 질병인자를 구분해 내고, 사람들 간 전염병의 전염경로를 추적하는 것은 물론 전염병의 전파를 예측할 수 있었음을 보고했는데, 수집된 트윗(1600만건) 중 GPS, 즉 위치가 태그된 트윗(약 450만건)을 분석 대상으로 삼고, 질병관련 단어가 들어가 있는 트윗을 추출해 질병인자를 갖고 있는 사람들이 만나는 위치(co-location) 및 사회 관계망(친구의 수)과 질병에 걸리는 확률간의 상관관계를 도출하였다.

한편, 32년간의 사회관계망 데이터를 수집하여 지난 30년간 비만이 매우 증가했고, 이 원인 중 하나로 사람 대 사람 간 비만의 전파가 있다는 것을 설득력 있게 설명하는 연구 [3]에서는 특정 지역의 총 12,067명의 BMI(Body-Mass Index) 지수를 1971년부터 2003년까지 빠짐없이 수집하여 그들의 체중 변화가 친구나 가족, 혹은 이웃 등 그들 주변 인물들의 체중변화에 영향을 받았는지 여부를 분석한 바 있다.

본론

■ 언론과 미디어에서의 건강정보 유통과 환경의 변화

우리나라의 경우 대부분의 사람들이 건강관련 정보를 매스미디어를 통해 주로 얻고 있는 반면, 젊은 세대 대다수는 인터넷을 통해 건강정보를 얻고 있다. 특히, SNS가 전통적인 언론의 의제 지배적인 역할을 무너뜨리면서 누구나 매체를 소유하고 의견을 표명할 수 있는 기회를 가지게 되었고, 소통채널 또한 다양해짐에 따라 의료 환경에 있어서도 질병 및 치료법들에 대한 사람들의 자유로운 공유가 이루어지고 있다. 이러한 현상은 의학2.0 (Medicine 2.0)이라 불리며 해외의 경우 많은 주목을 받고 있으며 애스크어페이션트(askapatient.com)와 구글의 독감예측(Google Flu)과 같은 실질적인 서비스로 발전하기도 하였다. 반면 SNS를 통한 정보의 유통은 인터넷 정보의 신뢰성 문제와 더불어 관련 이슈에 대한 언론보도의 내용과 SNS상의 정보에 대한 불일치, 찬반논란, 오정보, 괴담 등 수많은 문제를 야기하고 있으며, 국민들의 건강 관련 지식을 오도할 가능성이 충분하다. 따라서 이에 대한 면밀한 온라인 상 건강에 대한 정보공유와 담론에 대한 모니터링이 절실히 필요한 상황이다. 단 SNS 플랫폼의 정보 공유 형태에 따라 개방형 미디어의 경우 모니터링이 상대적으로 손쉽게 가능한 반면, 폐쇄형 미디어의 경우 데이터의 접근은 제한이 되는 반면, 전국민적인 이슈나 담론의 경우 폐쇄형 미디어와 개방형 미디어의 토픽이 상호 연관되는 점을 활용 가능하다.

■ 건강과 관련한 인문·자연환경(공간)의 영향과 질병연구

공간적 분석은 개인의 건강관련 지표나 행태자료, 그리고 다양한 환경요인을 공간적으로 표시하고 이들의 연관성을 기술하고 분석한다. 대표적인 연구주제는 질병과 관련한 환경의 영향, 건강 수준의 지역별 격차, 건강 서비스의 공간적 불균형, 건강 서비스에 대한 접근성 등을 포괄한다. 도시의 인구 사회학적 특성은 공간에 따라 분화되어 있기 때문에서 지역에 따라 커뮤니케이션의 내용과 미디어가 차별적으로 나타날 수 있음 것으로 기대되는데 [4], 질병 역시 마

찬가지로 단순히 개인의 건강 여부를 나타내는 것만이 아니라, 그 이면에 개인의 특성과 인문, 자연 환경의 영향, 사회적 관계, 그리고 제도적 장치 등 여러 인구사회학적 변수의 총체적 함수이며, 질병은 이 요인들이 복합적으로 작용하여 나타나는 공간적 현상으로 설명할 수 있다 [5]. 최신의 커뮤니케이션 미디어는 공간적 속성을 가지고 있는데 [6], 온라인 맵핑 등을 통해서 지리정보 자체가 미디어로 이용되며, SNS상에 위치 정보가 추가되면서 네트워크 서비스 등의 미디어가 공간정보의 속성을 가지게 된다. 점진적으로 미디어와 공간정보의 결합이 진행되고 있으며, 공간과 커뮤니케이션의 상호작용은 확대되고 있다. 따라서, 발암성 요인과 관련한 사회적 차원의 리스크 커뮤니케이션을 위해서는 단순한 한 두 가지의 방법론과 이론으로 설명되는 것이 아니라 개인의 인구 사회학적 변수에 공간적 환경을 포괄하는 다양한 측면의 분석이 요구된다. 다양한 공간 및 주변 환경의 영향을 파악하기 위해서는 개인의 이동과정 및 경로를 분석하여 이것이 리스크 커뮤니케이션에 어떻게 영향을 미치는지 확인할 필요가 있다.

■ 발암 관련 빅데이터 구축, 관리 시스템

기존의 발암관련 데이터는 여러 기관에 분산되어 있어, 데이터 간의 상관관계를 파악하기 어려운 점이 있으나 다양한 기관의 발암관련 데이터가 통합되어 분석되어야만 유용한 결과물을 도출할 수 있다. 이는 의료 및 보건과 관련된 정부 및 사설 의료 기관의 데이터에서 발암 리스크와 관련된 통계 데이터의 취합이 되어야 함을 의미한다. 아울러 효과적인 커뮤니케이션 관리를 위해서는 발암관련 메시지를 생산하고, 유통하는 미디어에 대한 포괄적인 데이터뿐만 아니라, 메시지를 생산하고 소비하는 개개인에 대한 데이터가 서로 유기적으로 연결되어 구축되어야 한다. 이 과정에서 기존 의료기관과 정부의 정형화된 데이터 뿐만 아니라 분석 대상인 미디어는 신문과 방송뿐 아니라 인터넷과 SNS를 포함하는 비정형 데이터도 다뤄야 한다. 발암성 위험 요인과 관련한 커뮤니케이션 내용과 사회적 계층, 연령 등에 따른 커뮤니케이션의 확산 및 전파과정을 공간적 맥락을 고려하여 시각적으로 구성하여 원활하고 효과적인 커뮤니케이션을 지원하는 것을 모색해야 한다.

■ 통합적이고 지속적인 패널연구

국민들의 암 관련 정보에 대한 이해도를 정확하게 파악하고, 하나의 이슈가 발생했을 때 사람들이 어떻게 인식하고 대처하는가에 대한 즉각적인 조사를 할 수 있는 발암원인 및 암 예방인식, 행태 관련 국민인식 패널조사체계가 구축되어야 할 필요가 있다. 이를 통해 시간의 흐름에 따른 국민들의 발암요인에 대한 인식과 지식, 태도의 변화를 추적조사 할 수 있고 잘못된 인식과 부적절한 정보의 유통에 대한 즉각적인 수정 조치를 할 수 있을 것으로 기대된다.

이러한 필요성에 기반하여 다학제의 최신 방법론을 적용한 발암성 위험 요인의 통합 빅데이터 구축을 중점으로 데이터 수집 및 저장, 관리에서부터 분석, 결과, 대국민 정보 전달에 이르는 일련의 과정을 위해 지속 가능한 리스크 커뮤니케이션 플랫폼 구축을 다음(그림 1)과 같이 제안해 볼 수 있다. 이러한 통합 플랫폼을 구축하고 국가 지정 기관을 통해 통합 시스템으로 관리함으로써 상황별 발암성 위험 요인 이슈 및 기타 건강관련 이슈에 특화된 정책 소통 방안을 시의적절하게 고안하고, 상황적 필요에 따라서 대응 전략 수립하며 세분화되고 최적화된 의사결정을 지원하는 데 기여할 수 있을 것으로 기대한다.

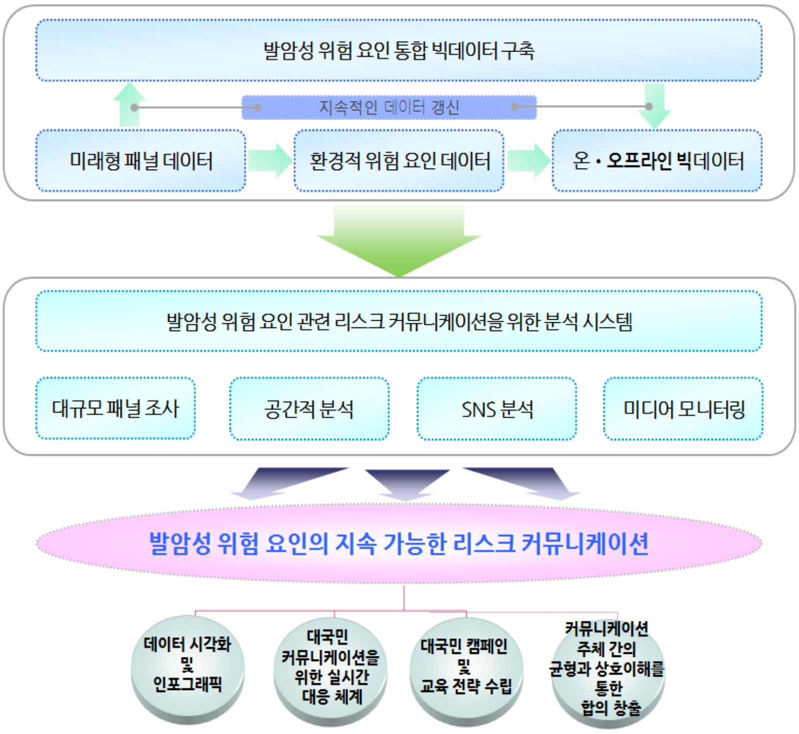

그림 1. 발암성 위험 요인 관련 지속 가능한 리스크 커뮤니케이션 플랫폼

발암성 위험 요인 관련 지속 가능한 리스크 커뮤니케이션 플랫폼 구축을 위해 필요한 과제들은 다음과 같이 제시해 볼 수 있다.

- 발암성 위험 요인 관련 통합 빅데이터 구축 및 관리

① 국가 암등록 데이터

국가 암등록 데이터는 암에 대한 체계적인 관리 및 암 연구에 대한 근거 자료로 제공하기 위하여 구축되고 있다. 암 환자의 지리적 분포는 암의 역학적 분석 상 중요한 요소 중 하나이므로 국가 암등록 데이터를 이용한 암 환자의 지리적 분석은 발암성 위험 요인 관련 중요한 자료로 사용될 수 있다.

② 인구관련 데이터

인구학적인 분석은 인문·사회 현상의 기반이 되는 연구로, 인구수, 인구 밀도, 인구 이동,

성비 및 연령 등이 인구학의 연구 범위에 포함되어 있다. 이렇게 구축된 인구학적 자료는 다양한 분야에서 응용이 가능하며, 공간의 속성 파악에도 효과적으로 사용될 수 있고, 특정 지역 내 사회·경제적 특징은 인구학적인 특징이 공간에 발현되어 나타나는 것이므로 인구 데이터와 함께 연구되어야 할 필요성이 있다. 인구학적인 데이터 외에도 직종, 소득, 학력과 지방세 등 사회·경제적 정보를 활용한 공간적 분석이 같이 이루어져야 한다. 국토교통부는 국가지리정보서비스를 통해 공간정보서비스, 국가공간정보통합, 전자국토서비스, 국가지리정보유통망 등 다양한 국토 및 교통 관련 통계자료와 GIS서비스를 통한 지도를 제공하고 있는데, 특히, 전국 공동주택 정보와 표준지공시지가, 부동산 정보 데이터는 지역 내 소득 분위의 추정에 필요한 자료로 활용될 수 있다. 이 밖에도, 통계청, 국토 교통부, 교육부에서 제공하는 통계자료와 인구 분석 기관에서 발행된 보고서를 통해 자료를 수집하고 필요한 정보를 선별하고 및 취합하는 작업이 요구된다.

③ 자연환경 데이터

자연 환경 데이터는 행동, 생활 양식 등 인간의 생활에 영향을 미치는 물리적 요소의 총체라고 할 수 있다. 고도나 경사, 기복, 토양 및 토지 피복 상태와 같은 지형의 상태나 연평균기온, 연교차, 강수, 바람, 습도 등 기후 요소 등이 자연 환경 데이터에 포함될 수 있다. 자연 환경 데이터의 개별 요소는 복합적으로 작용하기 때문에 개별 현상의 분석보다 종합적인 파악이 중요하다. 국토지리정보원에서는 수치지형도 및 토지 특성도, 토지이용현황도와 항공사진 영상 등을 관리 및 판매하고 있으며, 환경부에서는 환경부 산하 기관인 환경 지리 정보 서비스를 통해 토지 피복 현황 및 지표수 수질, 대기오염 및 폐기물 발생 현황 데이터를 제공하고 있다. 특히, 공공기관 사용자들에게는 환경공간정보자료의 원본자료 다운로드 서비스를 제공하고 있어 연구 진행 시 환경부에서 얻을 수 있는 자연환경 데이터의 수집이 용이할 것으로 기대된다. 기상청에서는 연평균 기온, 연교차, 강수, 풍속 및 풍향, 습도, 증발량, 서리일수, 안개일수 등 기상과 기후에 관련된 지도와 함께 매월 기후 통계 분석 자료를 제공하고 있다. 특히, 기상청에서 제공하는 기후 요소에 대한 통계자료와 기상 관측지 위치 포인트 데이터는 수집된 자료를 바탕으로 2차적인 가공이

가능하다. 이러한 자연 환경 데이터를 통합적인 분석이 가능하도록 변환하여, 인구 및 기타 공간 요소와 함께 암의 공간적 분석 연구에 활용할 수 있다.

④ 발암의 환경적 위험 요인 데이터

환경적 발암 물질이 자연적으로 존재하거나 발암물질이 인위적으로 방출되고 있다고 의심되는 시설이 있는 경우 주소 등 지리적 정보를 수집한 후 포인트 데이터의 구축이 필요하다. 환경적 발암 물질의 경우 우리나라에서는 통합적인 정의 및 리스트가 존재하지 않는다. 서로 일치하지 않는 내용을 담고 있지만, 노동부의 산업안전보건법과 환경부의 유해화학물질관리법에 기초하여 발암물질과 환경적 발암물질을 추출하는 것이 가능하다. 필요에 따라서 WHO 산하의 국제암연구소(IARC: International Agency for Research on Cancer)와 같은 외국 기관에서 작성한 발암물질 리스트를 사용할 수도 있다. 환경적 발암 물질의 위치 정보를 포인트 데이터로 기록하고 데이터 베이스로 구축하는 작업은 발암 관련 시설물에 대한 의심에 기초하기 때문에 많은 데이터의 신뢰성을 높이기 위한 후속 작업이 요구된다.

⑤ 구축된 포인트 데이터에는 산업 시설의 종류 및 발암 인자 방출량 및 영향력 등의 정보를 입력하여 발암과 관련한 분석에 활용할 수 있도록 한다. 발암의 환경적 위험요소는 시간에 따라 계속 변화하기 때문에 자료 수집 주기를 선정하여 계속적인 최신화가 필요하다. 발암의 환경적 위험 요인 데이터가 수집되고 나면 발암 물질이 배출되는 지점의 파악과 확산 속도, 영향력 등의 분석을 통해 해당 환경이나 시설이 주변 인구에 미치는 영향에 대한 분석이 가능하게 된다.

.

⑥ 발암성 위험 요인 관련 뉴스 모니터링

발암성 위험 요인에 대한 보도가 어떤 방식으로 이루어지고 있는지 추적하는 것은 매우 중요하다. 발암성 위험 요인에 대한 국민 인식은 상당한 정도로 언론 뉴스에 위해 영향을

받고 있기 때문이다. 뉴스 모니터링 결과는 다른 데이터들 (예를 들어 SNS 담론 데이터) 등과 통합되어 발암성 위험 요인에 대한 사회적 담론이 어떻게 확산되는지 경로를 추적하고 분석하는 과정에서 사용된다. 또한 언론 자체를 통합적 위기 관리 시스템의 중요한 주체로 만드는 제도 구축을 위해서도 뉴스 모니터링 결과는 중요한 데이터를 제공할 것이다.

발암성 위험 요인 관련 뉴스 모니터링을 위한 분석대상은 서울과 주요 광역시의 대표적 뉴스매체들(신문과 방송), 10대 주요 일간지, 방송 채널(KBS, MBC, SBS 이외에도 서울 및 주요 광역시의 대표적인 방송을 사전조사를 통해 선별)이다. 데이터 수집은 한국언론재단의 언론보도 데이터베이스를 이용하며 분석 데이터 선별은 분석 대상이 되는 매체 중 키워드 '발암', '발암보도', '발암정보'로 검색되는 모든 기사를 전수 조사한다. 주요 분석 항목은 보도량, 보도 프레임, 보도의 태도와 방향, 보도의 질 등이 해당된다.

⑦ 발암성 위험 요인 관련 SNS상의 담론 분석

SNS상의 대용량 데이터 수집 및 정리를 위해, 발암 요인과 관련된 다양한 담론 및 사용자들을 수집할 수 있는 최적의 사회 관계망 데이터 마이닝의 방법 및 기간, 범위를 설정한다. 이렇게 모아진 대용량의 SNS 데이터를 빠르게 처리하고 분석하기 위해 데이터 베이스화 하는 작업을 진행한 후, 발암 요인들과 관련된 단어들을 선정하여 이를 SNS에 적용하기 알맞은 상태로 수정한다. 근거 기반의 발암 요인들을 선정하고, SNS 컨텐츠의 검색에 사용될 연관 검색어를 추가 선정한다. SNS 데이터에서 발암 관련 단어들을 언급한 컨텐츠 및 사용자들만을 뽑아내어 이들의 사회 관계망적 특성을 다양한 양적 분석 기법들을 통해 살펴본다. 각종 발암 요소들의 특성에 따라 이를 언급하는 사용자들의 사회 관계망은 어떻게 달라지는지, 혹은 그 요소들에 관한 잘못된 정보를 퍼뜨리는 사용자들의 관계망은 어떻게 달라지는지를 사회 관계망의 밀도(density), 중심성(centrality), 군집 계수(clustering coefficient), 구조적 공백(structural hole)과 같은 양적 지표들의 비교를 통해 살펴볼 수 있다. 암의 종류에 따라 SNS 내에 각 암에 관해 언급한 사용자들의 이러한 사회 관계망 관련 양적 지표들이 어떻게 달라지는지도 확인한다. 양적 분석을 마친 후, 자연어 분석 기법들을 이용하여 SNS 내 발암 관련 담론들의 감성을 분석하게 된다. 이를 통해 다음과 같은 다양한 질적 연구 주제들도 도

출될 수 있다.

- 각종 발암 물질 및 요인들에 관련한 뉴스나 정보들에 대해 사람들은 어떻게 감정으로 반응하는가?
- 이러한 감정은 해당 정보가 거짓으로 밝혀졌을 때와 과학적인 실험을 통해 사실로 검증되었을 때 어떻게 변화해 가는가?
- 특정 음식이 발암 물질을 함유한 것으로 새로운 정보가 나타났을 때 사람들의 감정은 얼마나 빨리, 어떠한 감정 상태로 변화해 가는가?
- 더불어, 컴퓨터를 이용한 자연어 분석 기법이 가지는 정확성의 문제를 극복하기 위해 SNS 컨텐츠를 직접 읽고 내용을 파악 및 분석하는 고전적인 방법들도 보완적으로 사용될 수 있다. 특히, 최근에 많은 질적 연구들에서 활발하게 사용되고 있는 아마존(Amazon) 사의 미케니컬 터크(Mechanical Turk)라는 서비스를 이용, 적은 비용으로 다양한 질적 수작업들을 보완적으로 병행하여 분석을 진행할 수 있다. 국내의 경우 규모는 작지만 설문조사 기반의 유사 서비스가 존재하고 있고, 인터넷 보급률과 참여율이 높은 국내 상황을 고려하여 위키(wiki)기반의 토픽분석도 가능하리라 기대한다.

⑧ GIS를 활용한 SNS 빅데이터 기반의 공간적 분석

인문, 사회, 자연 환경을 나타낼 수 있는 공간 데이터로서 토지 이용, 교통로, 지하철, 버스, 공원, 병원수, 교육기관 수 등이 포함되며, 자연 환경 데이터로서 하천, 산, 녹지 등이 포함되고 기초 통계 정보로서 행정구역, 사업체 수 및 종사자 수, 인구학적 특성, 전출입 정보, 지방세 합계액 등이 포함된다. 이 외에 필요한 경우 특정 지역에 대하여 설문조사를 실시한다. 성별, 연령, 직업, 거주인구의 비율을 반영한 설문 구역 및 대상을 할당하고 설문자의 스마트폰에 조사용 어플리케이션을 설치한다. 설문 조사 후 설문 내용의 특성과 공간적 분포에 따른 지역적 편차와 인구 사회적 주변 환경의 상관관계를 분석하게 된다. 설문대상자의 시공간적 이동패턴을 파악하기 위한 스마트폰 앱은 일정 시간마다 스마트 폰 이용자의 위치정보를 서버로 전송하며

서버는 시간의 흐름에 따른 개인의 위치 정보를 기록한다. 성별, 연령, 직업 등으로 구분한 이용자의 주요 시간대 별 근로시간, 여가시간, 가정 체류시간에 따른 활동 지역을 군집 분류하고, 이에 따른 리스크 커뮤니케이션의 내용을 비교하게 된다. 개인의 이동 경로에 따라 환경요인에 대한 노출 평가를 할 수 있으며 GPS/GIS 기술을 이용한 분석은 환경, 인문 사회적 위험 요인에 대한 정확한 평가를 가능하게 한다.

⑨ 발암 요인에 대한 국민 인식 패널 조사

국민들의 암 관련 정보에 대한 이해도를 정확하게 파악하고, 하나의 이슈가 발생했을 때 사람들이 어떻게 인식하고 대처하는가에 대한 조사를 하기 위해서는 발암원인 및 암 예방 인식, 행태 관련 국민인식 패널조사체계가 구축되어야 할 필요가 있다. 이를 통해 시간의 흐름에 따른 국민들의 발암요인에 대한 인식과 지식, 태도의 변화를 추적조사 할 수 있고 잘못된 인식과 부적절한 정보의 유통에 대한 즉각적인 수정 조치를 할 수 있을 것으로 기대된다.

주요 조사 문항으로는 암 유발 요인에 대한 지식수준, 태도와 신념, 정보 추구 동기, 행동 패턴, 정보 획득원 및 정보 공유패턴 등을 조사하게 되며, 이를 통해 발암 요인에 대한 국민들의 지식 수준 정도, 발암 요인 인식에 있어서의 차이를 만들어내는 개인적, 사회네트워크적, 지역적 수준의 요인들, 발암 요인에 대한 지식과 정보의 취약 계층, 발암 요인과 관련한 루머의 확산 정도, 발암 요인에 대한 지식과 예방 행동 사이의 관계, 발암 요인에 대한 잘못된 인식과 예방 행동 사이의 관계 등을 규명하며, 나아가 발암 요인과 관련한 루머 등 오정보에 취약한 계층을 대상으로 한 효과적인 중재방안을 모색하는 데 중요한 자료로 활용될 것이다.

⑩ 발암성 위험 요인 관련 SNS 담론에 참여한 사용자들에 대한 모니터링과 심층 인터뷰 실시

발암 요인 및 물질과 관련하여 온라인상에서 SNS 담론에 참여한 사용자들에 대해 모니터링과 심층 인터뷰를 실시한다. SNS 데이터 수집 시 얻게 된 사용자의 SNS 아이디를 이용, 특정 기간에 걸쳐 대상 사용자들의 SNS 사용 및 다른 이슈들에 대한 반응들을 살펴보고 이를 이전의 발암 관련 반응들과 비교해볼 수 있다. 또한, 발암 물질 및 요인과 관련하여 잘못된

정보를 올리거나 전파하는 사람들을 모니터링 하며 다른 이슈들에 대한 그들의 SNS 이용 행태를 관찰하고 비교한다. 이러한 이슈별, 그룹별, 시기별 다각도의 분석을 마친 후 결과의 최종 해석을 위해 이용자들 중 샘플 그룹을 선정 심층 인터뷰를 실시 한 후, 이를 통해 SNS 컨텐츠 분석만으로는 찾아내기 힘들었던, 잘못된 정보 전파의 동기나 담론에 참여하고 정보를 전하거나 전파하는 사용자들의 의학적 전문성, 주로 그들이 정보를 얻는 오프라인의 정보원 등의 자료를 얻을 수 있을 것으로 기대된다.

⑪ 효과적인 리스크 커뮤니케이션을 위한 데이터 시각화·인포그래픽 구현

최근 인포그래픽의 가치는 미디어, 기업뿐만 아니라, 공공 차원(정부, 기관, 학교 등)에서도 차츰 확산되고 있는 추세이며, 요즘은 공공기관의 정보전달의 일환으로 대국민 서비스와 보도자료도 인포그래픽의 형태로 업그레이드 되고 있다. 빅데이터 분석 결과 제시 방법에서 데이터 시각화 과정은 필수적이라고 판단되며, 시각화 및 인포그래픽 구현작업은 사회적 논란이 되는 위험요인이나 이슈에 대해 일반인 집단에게 효과적이며 이해하기 쉽게 전달할 수 있는 설득력 있는 툴이 될 수 있다

결론

상기에서 제시한 발암성 위험 요인 관련 지속 가능한 리스크 커뮤니케이션 플랫폼은 발암 관련 이슈와 관련해서 발생하는 다양한 여론 흐름에 대한 단기적, 장기적인 대응책을 마련하는데 도움이 될 것으로 생각한다. 단기적으로는 발암성 위험 요인에 대한 특정 이슈가 발생했을 때 그에 대한 대국민 홍보 체계를 수립하는데 사용될 수 있고, 장기적으로는 한국 사회에서의 암 관련 정보역학(infodemiology) [7]을 위한 포괄적인 데이터를 축적할 수 있을 것으로 생각한다. 또한, 다학제적 융합연구를 통해 각 학제간의 방법론적 제한점을 극복하는 기술적인 인프라 구축에도 기여할 것으로 기대한다.

참고문헌

1.Jung K-W, Won Y-J, Kong H-J, Oh C-M, Lee DH, Lee JS. Cancer Statistics in Korea: Incidence, Mortality, Survival, and Prevalence in 2011. Cancer Research and Treatment 2014;46:109-123.
2.Sadilek A, Kautz H, Silenzio V. Modeling Spread of Disease from Social Interactions; 2012
3.Christakis NA, Fowler JH. The Spread of Obesity in a Large Social Network over 32 Years. New England Journal of Medicine 2007;357:370-379.
4.Adams PC. Geogrpahies of Media and Communication. Malden: Wiley-Blackwell; 2009
5.Dubos R. Man adapting. New Haven: Yale University Press; 1965
6.Sui D, Goodchild M. The convergence of GIS and social media: challenges for GIScience. International Journal of Geographical Information Science 2011;25:1737-1748.
7.Eysenbach G. Infodemiology and infoveillance: framework for an emerging set of public health informatics methods to analyze search, communication and publication behavior on the Internet. Journal of medical Internet research 2009;11
